# *N*,*N*′-Bis(2-hydroxy­ethyl)benzene-1,4-dicarboxamide

**DOI:** 10.1107/S1600536808017467

**Published:** 2008-06-19

**Authors:** Gabriela Bednarek, Joachim Kusz, Alicja Ratuszna, Jerzy Ossowski, Wiesław W. Sułkowski

**Affiliations:** a Institute of Physics, University of Silesia, 40-007 Katowice, Poland; b Department of Environmental Chemistry and Technology, Institute of Chemistry, University of Silesia, 40-007 Katowice, Poland

## Abstract

The mol­ecule of the title compound, C_12_H_16_N_2_O_4_, is centrosymmetric and the amide group is twisted relative to the benzene ring by 14.40 (13)°. The mol­ecules are hydrogen bonded into a three-dimensional framework, with the hydr­oxy O atoms acting as acceptors in N—H⋯O hydrogen bonds and as donors in O—H⋯O=C inter­actions.

## Related literature

For the synthesis of the title compound, see: Sułkowski *et al.* (2000 ▶); Shukla & Harad (2006 ▶). For bond-length data, see: Allen (2002 ▶). For hydrogen bonding, see: Desiraju & Steiner (1999 ▶).
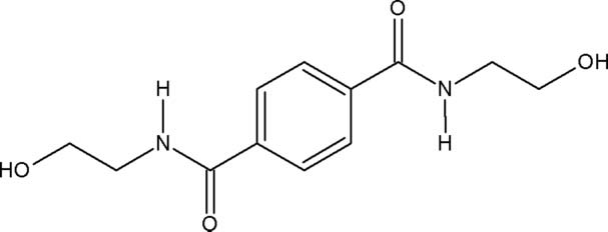


## Experimental

### 

#### Crystal data

C_12_H_16_N_2_O_4_
*M* *_r_* = 252.27
Monoclinic, 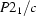

*a* = 4.9062 (4) Å
*b* = 13.6467 (10) Å
*c* = 8.8840 (7) Å
β = 97.262 (6)°
*V* = 590.04 (8) Å^3^
*Z* = 2
Mo *K*α radiation
μ = 0.11 mm^−1^
*T* = 200 (1) K
0.26 × 0.22 × 0.18 mm

#### Data collection

Oxford Diffraction KM-4-CCD Sapphire3 diffractometer
Absorption correction: none
5655 measured reflections
2000 independent reflections
1599 reflections with *I* > 2σ(*I*)
*R*_int_ = 0.013

#### Refinement

*R*[*F* ^2^ > 2σ(*F* ^2^)] = 0.035
*wR*(*F* ^2^) = 0.097
*S* = 1.02
2000 reflections
114 parameters
All H-atom parameters refined
Δρ_max_ = 0.34 e Å^−3^
Δρ_min_ = −0.21 e Å^−3^

### 

Data collection: *CrysAlis CCD* (Oxford Diffraction, 2006 ▶); cell refinement: *CrysAlis RED* (Oxford Diffraction, 2006 ▶); data reduction: *CrysAlis RED*; program(s) used to solve structure: *SHELXS97* (Sheldrick, 2008 ▶); program(s) used to refine structure: *SHELXL97* (Sheldrick, 2008 ▶); molecular graphics: *PLATON* (Spek, 2003 ▶) and *Mercury* (Macrae *et al.*, 2006 ▶); software used to prepare material for publication: *SHELXL97*.

## Supplementary Material

Crystal structure: contains datablocks I, global. DOI: 10.1107/S1600536808017467/gk2148sup1.cif

Structure factors: contains datablocks I. DOI: 10.1107/S1600536808017467/gk2148Isup2.hkl

Additional supplementary materials:  crystallographic information; 3D view; checkCIF report

## Figures and Tables

**Table 1:** Hydrogen-bond geometry (Å, °)

*D*—H⋯*A*	*D*—H	H⋯*A*	*D*⋯*A*	*D*—H⋯*A*
N1—H3⋯O2^i^	0.879 (16)	2.080 (16)	2.9333 (10)	163.3 (13)
O2—H8⋯O1^ii^	0.863 (18)	1.872 (18)	2.7204 (9)	167.1 (15)
C2—H2⋯O2^i^	0.972 (15)	2.412 (14)	3.3458 (11)	161.0 (11)
C5—H4⋯O1^ii^	0.988 (12)	2.523 (12)	3.2738 (12)	132.6 (9)
C5—H5⋯O1^iii^	0.992 (13)	2.612 (13)	3.5671 (12)	161.7 (11)

Symmetry codes: (i) 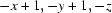; (ii) 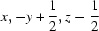; (iii) 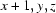.

**Fig. 1.**
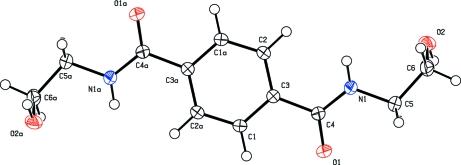
Molecular structure of the title compound. Displacement ellipsoids are drawn at the 50% probability level. The H-atom radius is arbitrary. Symmetry code: (a) -x, -y + 1, -z + 1

**Fig. 2.**
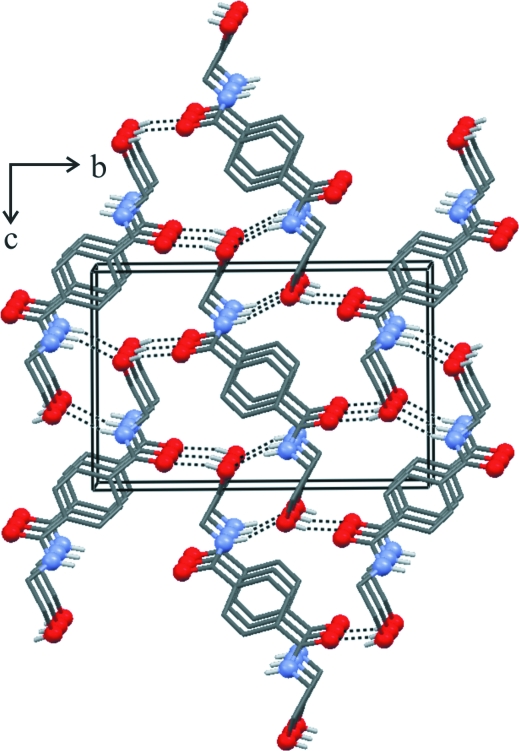
Packing diagram for the title compound. Hydrogen bonds are shown with dashed lines. Hydrogen atoms not involved in hydrogen bonding are omitted for clarity.

## supplementary crystallographic information

### Comment

Polyethylene terephthalate (PET) is a very popular thermoplastic polyester. The chemical recycling of its wastes has been the subject of keen interest as a valuable material for different chemical processes. Aminolysis of PET yields N, N' - bis(2-hydroxyethyl)benzene-1,4-dicarboxamide, which can be a potential candidate for further reactions leading to obtain other useful products. To get information about the hydrogen bonding in this interesting material we determined its crystal structure. In crystal the title molecule is located around inversion center (Fig. 1.).

The value of the C2—C3—C4 angle of 123.58 (7)° is in agreement with a geometry of the Ph—C(=O)—NH—CH_2 _subunit. A search of the Cambridge Structural Database [version 5.28; Allen, 2002] shows that in similar compounds this angle is consistently greater than 120° with the mean value of 122.46 (8)°. The widening of this angle can be related to a steric hindrance between H3 of the amide group and H atom attached to C2, as the consequence of a small twist of the amide group relative to the benzene ring. The torsion angles around the C—C bond between the amide group and the benzene ring are: C1—C3—C4—O1 14.40 (13)° and C2—C3—C4—N1 14.74 (13)°.

The molecules of the title compound are connected *via* N—H···O, O—H···O and C—H···O hydrogen bonds (Fig. 2; Table 1) into a three-dimensional framework. All N and O atoms participate in hydrogen bonding. The IR spectrum of the title compound shows bands corresponding to the N—H and O—H stretching vibrations in the 3370 - 2480 cm^-1^ region. The center of gravity of the ν_N—H_ and ν_O—H_ bands is located at *ca* 2960 cm^-1^. The relative shifts of about 440 cm^-1^ and 640 cm^-1^ for N—H and O—H bands allow to classify the N—H···O and O—H···O interactions in this crystal as strong hydrogen bonds (Desiraju & Steiner, 1999).

### Experimental

The title compound was obtained according to the method described by Sułkowski *et al.* (2000) and Shukla & Harad (2006). Single crystal suitable for X-ray analysis was obtained from water solution. Analysis calculated: C 57.13, H 6.39, N 11.10%; found C 57.12, H 6.26, N 10.93%. IR spectra were recorded with the Perkin-Elmer Spectrum.

### Refinement

All H atoms were located in a difference Fourier map and freely refined with isotropic displacement parameters.

### Crystal data

**Table:**

C_12_H_16_N_2_O_4_	*F*_000_ = 268
*M**_r_* = 252.27	*D*_x_ = 1.420 Mg m^−^^3^
Monoclinic, *P*2_1_/*c*	Mo *K*α radiation λ = 0.71073 Å
Hall symbol: -P 2ybc	Cell parameters from 3594 reflections
*a* = 4.9062 (4) Å	θ = 3.0–32.8º
*b* = 13.6467 (10) Å	µ = 0.11 mm^−^^1^
*c* = 8.8840 (7) Å	*T* = 200 (1) K
β = 97.262 (6)º	Needle, colourless
*V* = 590.04 (8) Å^3^	0.26 × 0.22 × 0.18 mm
*Z* = 2	

### Data collection

**Table:**

Oxford Diffraction KM-4-CCD Sapphire3 diffractometer	2000 independent reflections
Radiation source: fine-focus sealed tube	1599 reflections with *I* > 2σ(*I*)
Monochromator: graphite	*R*_int_ = 0.014
Detector resolution: 16.0328 pixels mm^-1^	θ_max_ = 32.9º
*T* = 200(1) K	θ_min_ = 3.0º
ω scans	*h* = −7→5
Absorption correction: none	*k* = −19→19
5655 measured reflections	*l* = −13→12

### Refinement

**Table:**

Refinement on *F*^2^	Secondary atom site location: difference Fourier map
Least-squares matrix: full	Hydrogen site location: inferred from neighbouring sites
*R*[*F*^2^ > 2σ(*F*^2^)] = 0.035	All H-atom parameters refined
*wR*(*F*^2^) = 0.097	*w* = 1/[σ^2^(*F*_o_^2^) + (0.0542*P*)^2^ + 0.0884*P*] where *P* = (*F*_o_^2^ + 2*F*_c_^2^)/3
*S* = 1.03	(Δ/σ)_max_ < 0.001
2000 reflections	Δρ_max_ = 0.34 e Å^−^^3^
114 parameters	Δρ_min_ = −0.21 e Å^−^^3^
Primary atom site location: structure-invariant direct methods	Extinction correction: none

### Special details

**Table:**

Geometry. All e.s.d.'s (except the e.s.d. in the dihedral angle between two l.s. planes) are estimated using the full covariance matrix. The cell e.s.d.'s are taken into account individually in the estimation of e.s.d.'s in distances, angles and torsion angles; correlations between e.s.d.'s in cell parameters are only used when they are defined by crystal symmetry. An approximate (isotropic) treatment of cell e.s.d.'s is used for estimating e.s.d.'s involving l.s. planes.
Refinement. Refinement of *F*^2^ against ALL reflections. The weighted *R*-factor *wR* and goodness of fit *S* are based on *F*^2^, conventional *R*-factors *R* are based on *F*, with *F* set to zero for negative *F*^2^. The threshold expression of *F*^2^ > σ(*F*^2^) is used only for calculating *R*-factors(gt) *etc*. and is not relevant to the choice of reflections for refinement. *R*-factors based on *F*^2^ are statistically about twice as large as those based on *F*, and *R*- factors based on ALL data will be even larger.

### Fractional atomic coordinates and isotropic or equivalent isotropic displacement parameters (Å^2^)

**Table:**

	*x*	*y*	*z*	*U*_iso_*/*U*_eq_	
O1	0.27008 (15)	0.27588 (5)	0.35222 (8)	0.03059 (17)	
H8	0.422 (3)	0.3500 (13)	−0.1314 (17)	0.051 (4)*	
O2	0.50462 (15)	0.40412 (5)	−0.10308 (8)	0.02923 (17)	
N1	0.45385 (15)	0.39818 (5)	0.22776 (8)	0.02234 (16)	
H3	0.470 (3)	0.4611 (12)	0.2100 (16)	0.043 (4)*	
C1	−0.0348 (2)	0.59732 (6)	0.45869 (11)	0.02646 (19)	
H1	−0.063 (3)	0.6650 (11)	0.4282 (15)	0.037 (3)*	
C2	0.1105 (2)	0.53512 (7)	0.37400 (10)	0.02685 (19)	
H2	0.183 (3)	0.5608 (11)	0.2851 (16)	0.044 (4)*	
C3	0.14642 (16)	0.43721 (6)	0.41503 (9)	0.01995 (16)	
C4	0.29698 (17)	0.36475 (6)	0.32869 (9)	0.02059 (16)	
C5	0.61451 (18)	0.33030 (6)	0.14753 (10)	0.02380 (17)	
H4	0.493 (2)	0.2750 (9)	0.1119 (13)	0.028 (3)*	
H5	0.772 (3)	0.3050 (10)	0.2180 (15)	0.037 (3)*	
C6	0.71991 (19)	0.37877 (7)	0.01299 (11)	0.02745 (19)	
H6	0.851 (3)	0.3331 (10)	−0.0254 (15)	0.039 (3)*	
H7	0.816 (2)	0.4390 (10)	0.0435 (14)	0.029 (3)*	

### Atomic displacement parameters (Å^2^)

**Table:**

	*U*^11^	*U*^22^	*U*^33^	*U*^12^	*U*^13^	*U*^23^
O1	0.0417 (4)	0.0180 (3)	0.0354 (4)	0.0064 (3)	0.0175 (3)	0.0056 (2)
O2	0.0438 (4)	0.0192 (3)	0.0259 (3)	−0.0063 (3)	0.0089 (3)	0.0005 (2)
N1	0.0275 (4)	0.0175 (3)	0.0238 (3)	0.0003 (2)	0.0099 (3)	−0.0013 (2)
C1	0.0365 (5)	0.0179 (4)	0.0276 (4)	0.0047 (3)	0.0145 (3)	0.0056 (3)
C2	0.0372 (5)	0.0203 (4)	0.0260 (4)	0.0041 (3)	0.0155 (3)	0.0052 (3)
C3	0.0229 (4)	0.0182 (3)	0.0196 (3)	0.0017 (3)	0.0057 (3)	0.0014 (3)
C4	0.0234 (3)	0.0188 (4)	0.0200 (3)	0.0025 (3)	0.0044 (3)	0.0014 (3)
C5	0.0270 (4)	0.0214 (4)	0.0243 (4)	0.0037 (3)	0.0083 (3)	−0.0013 (3)
C6	0.0288 (4)	0.0252 (4)	0.0310 (4)	−0.0020 (3)	0.0140 (3)	−0.0027 (3)

### Geometric parameters (Å, °)

**Table:**

O1—C4	1.2404 (10)	C3—C1^i^	1.3913 (12)
O2—H8	0.863 (18)	C3—C4	1.5017 (11)
N1—C4	1.3338 (11)	C5—H4	0.988 (12)
N1—C5	1.4594 (11)	C5—H5	0.992 (13)
N1—H3	0.879 (16)	C6—O2	1.4224 (12)
C1—H1	0.967 (14)	C6—C5	1.5133 (12)
C2—C1	1.3896 (12)	C6—H6	0.989 (14)
C2—C3	1.3902 (12)	C6—H7	0.970 (13)
C2—H2	0.972 (15)		
			
C3—C2—C1	120.12 (8)	C2—C3—C4	123.58 (7)
C3—C2—H2	120.8 (9)	C1^i^—C3—C4	117.25 (7)
C1—C2—H2	119.0 (9)	N1—C5—C6	111.58 (7)
O2—C6—C5	112.48 (7)	N1—C5—H4	107.5 (7)
O2—C6—H7	106.7 (7)	C6—C5—H4	109.4 (7)
C5—C6—H7	110.7 (7)	N1—C5—H5	109.7 (8)
O2—C6—H6	111.1 (8)	C6—C5—H5	109.6 (8)
C5—C6—H6	107.4 (8)	H4—C5—H5	108.9 (10)
H7—C6—H6	108.4 (10)	O1—C4—N1	122.04 (8)
C6—O2—H8	106.3 (10)	O1—C4—C3	119.21 (7)
C4—N1—C5	120.29 (7)	N1—C4—C3	118.75 (7)
C4—N1—H3	122.0 (9)	C2—C1—C3^i^	120.73 (8)
C5—N1—H3	117.7 (9)	C2—C1—H1	119.7 (8)
C2—C3—C1^i^	119.16 (7)	C3^i^—C1—H1	119.6 (8)
			
C1—C2—C3—C1^i^	0.09 (16)	C2—C3—C4—O1	−164.62 (9)
C1—C2—C3—C4	179.10 (8)	C1^i^—C3—C4—O1	14.40 (13)
C4—N1—C5—C6	165.93 (8)	C2—C3—C4—N1	14.74 (13)
O2—C6—C5—N1	−66.81 (10)	C1^i^—C3—C4—N1	−166.23 (8)
C5—N1—C4—O1	−3.87 (13)	C3—C2—C1—C3^i^	−0.09 (16)
C5—N1—C4—C3	176.78 (7)		

Symmetry codes: (i) −*x*, −*y*+1, −*z*+1.

### Hydrogen-bond geometry (Å, °)

**Table:**

*D*—H···*A*	*D*—H	H···*A*	*D*···*A*	*D*—H···*A*
N1—H3···O2^ii^	0.879 (16)	2.080 (16)	2.9333 (10)	163.3 (13)
O2—H8···O1^iii^	0.863 (18)	1.872 (18)	2.7204 (9)	167.1 (15)
C2—H2···O2^ii^	0.972 (15)	2.412 (14)	3.3458 (11)	161.0 (11)
C5—H4···O1^iii^	0.988 (12)	2.523 (12)	3.2738 (12)	132.6 (9)
C5—H5···O1^iv^	0.992 (13)	2.612 (13)	3.5671 (12)	161.7 (11)

Symmetry codes: (ii) −*x*+1, −*y*+1, −*z*; (iii) *x*, −*y*+1/2, *z*−1/2; (iv) *x*+1, *y*, *z*.
